# On a remarkable geometric-mechanical synergism based on a novel linear eigenvalue problem

**DOI:** 10.1007/s00707-021-03091-5

**Published:** 2021-11-19

**Authors:** Johannes Kalliauer, Michał Malendowski, Herbert A. Mang

**Affiliations:** 1 Institute for Mechanics of Materials and Structures, TU Wien – Technische Universität Wien, Karlsplatz 13/202, 1040 Wien, Austria; 2 Institute of Structural Analysis, Poznan University of Technology, Plac Marii Skłodowskiej-Curie 5, 60-965 Poznań, Poland; 3 Tongji University, College of Civil Engineering, Siping Road 1239, 200092 Shanghai, China

## Abstract

The vertices of two specific eigenvectors, obtained from a novel linear eigenvalue problem, describe two curves on the surface of an *N*-dimensional unit hypersphere. *N* denotes the number of degrees of freedom in the framework of structural analysis by the Finite Element Method. The radii of curvature of these two curves are 0 and 1. They correlate with pure stretching and pure bending, respectively, of structures. The two coefficient matrices of the eigenvalue problem are the tangent stiffness matrix at the load level considered and the one at the onset of loading. The goals of this paper are to report on the numerical verification of the aforesaid geometric-mechanical synergism and to summarize current attempts of its extension to combinations of stretching and bending of structures.

## Introduction

Generally, it is preferable that the loads are mainly carried by membrane and axial forces instead of bending moments. A measure to which extent this goal is reached is the percentage of the ”non-membrane” energy of the total strain energy. It is defined as ${({U-U_M})}/{U}$, where $U_M$ denotes the membrane (stretching) energy and *U* stands for the total strain energy. The lower bound of this ratio is zero, and it refers to pure stretching. The upper bound is one, and it refers to pure bending.

The basic idea of this work is to find a geometric quantity such that its lower and upper bound agrees with the bounds of ${({U-U_M})}/{U}$. This is done with the help of a novel linear eigenvalue problem in the framework of the Finite Element Method (FEM). Such a quantity is the radius of the first Frenet-curvature of a curve on the surface of an *N*-dimensional unit hypersphere, denoted as $\rho$. The reason why $\rho$ is associated with such a hypersphere is that the vertex of the unit vector, describing this curve, is an eigenvector of a novel linear eigenvalue problem referring to a structure with *N* degrees of freedom, discretized by the FEM. One of the two coefficient matrices of this eigenvalue problem is the tangent stiffness matrix. The other one needs to be determined such that the two aforementioned limiting values of $\rho$ can be realized for pure stretching and pure bending, respectively.

The significance of these two limiting cases of ${({U-U_M})}/{U}$ stems from *a priori* knowledge of the values of this ratio. Hence, there is no need to verify the hypothetically assumed correlation between ${({U-U_M})}/{U}$ and $\rho$ by computing *U* and $U_M$ separately by the FEM. If, however, an expanded form of the geometric-mechanical synergism described in this work could be shown to exist, which so far has not been the case, direct computation of ${({U-U_M})}/{U}$ would be advantageous. It is mentioned, in passing, that the deformations do not directly enter into the calculation of $\rho$.

The first forerunner of the present work was a paper by Mang et al. [8]. The linear eigenvalue problem used for the determination of $\rho$ was the so-called ”Consistently Linearized Eigenvalue Problem” (CLE). It was originally proposed by Helnwein [3]. The two coefficient matrices of this eigenvalue problem are the tangent stiffness matrix and its derivative with respect to a dimensionless load parameter in the framework of proportional loading. A disadvantage of this model was the explicit dependence of $\rho$ on the relevant eigenvalue, defined as the zero eigenvalue at the stability limit. This resulted in the vanishing of $\rho$ at the stability limit. Thus, the hypothesis $\rho ={({U-U_M})}/{U}$ [8] incorrectly signaled pure stretching at this point instead of pure bending. Another weak spot of the CLE was the *ab initio* indefiniteness of the derivative of the tangent stiffness matrix, resulting in positive as well as negative eigenvalues in the prebuckling domain and in conjugate complex eigenvalues when the tangent stiffness matrix became indefinite after the stability limit [16], notwithstanding the insignificance of the primary load-displacement path after this has happened. A numerical challenge of the CLE was a sufficiently accurate finite-difference approximation of the derivative of the tangent stiffness matrix [4, 9].

Another forerunner of the present work was a paper by Mang [6], characterized by the replacement of $\rho$ by $k\,a$ , where *a* denotes the acceleration of a fictitious particle moving along a curve on the surface of an *N*-dimensional unit hypersphere, obtained by the CLE, and where *k* stands for a proportionality factor. Although good numerical results were obtained for the two aforementioned limiting cases, in retrospect, lack of the invariance of $k\,a$ with respect to the chosen parameter is viewed as a shortcoming of that approach.

The present work is organized as follows: In Section 2, the FEM-based linear eigenvalue problem is introduced. One of its two coefficient matrices is the tangent stiffness matrix. The other one is yet to be determined. Herein, it is considered to be a constant, symmetric, positive definite matrix. Section 3 is devoted to the numerical implementation of the theoretical concept. This includes the numerical solution of the underlying eigenvalue problem and the numerical determination of $\rho$ with the help of finite-difference approximations of the first and the second derivative of the relevant eigenvector with respect to the parameter, to be defined in Section 2. Section 4 deals with the numerical verification of the asserted geometric-mechanical synergism for the limiting cases of pure stretching and pure bending. In Section 5, current attempts to numerically prove the existence of a wider range of validity of the geometric-mechanical synergism, delineated in this work, are described.

## Linear eigenvalue problem for the determination of $\rho$

The mathematical formulation of the chosen eigenvalue problem reads as

1 $$\begin{aligned} \left[ {\mathbf {K}}_T(\xi (\lambda ))-\chi (\xi (\lambda ))\,\mathbf {B}\right] \cdot \mathbf {r}(\xi (\lambda ))=\mathbf {0},\qquad \xi =\int _{\mathbf {q}=\mathbf {q}(0)}^{\mathbf {q}=\mathbf {q}(\lambda )}\left\| \mathrm d\mathbf {q}\right\| \,, \end{aligned}$$

where ${\mathbf {K}}_T$ is the tangent stiffness matrix in the framework of the FEM and $\mathbf {B}$ is a constant, symmetric, positive definite matrix. $\mathbf {B}$ must enable determination of the first eigenpair $(\chi _1,\mathbf {r}_1)$, with $\chi _1$ denoting the smallest eigenvalue and $\mathbf {r}_1$ standing for the corresponding eigenvector, normalized to 1, such that

2 $$\begin{aligned} \mathbf {r}_1(\xi (\lambda ))=\mathbf{const}. \end{aligned}$$

The parameter $\xi$ represents an arc length in the context of the FEM. It depends on the vector of differential node displacements, following from the equilibrium relation

3 $$\begin{aligned} {\mathbf {K}}_T\cdot \mathrm d\mathbf {q}=\mathrm d\lambda \,\widetilde{\mathbf {P}}\,, \end{aligned}$$

with $\widetilde{\mathbf {P}}$ standing for the vector of work-equivalent node forces. If snap-through can be ruled out, $\xi$ is replaced by the dimensionless load parameter $\lambda$.

Once $\mathbf {r}_1$ is known, $\rho _1$ can be computed from [2, 5, 14]

4 $$\begin{aligned} \rho _1=\frac{\left\| \dot{\mathbf {r}}_1\right\| ^3}{\sqrt{\dot{\mathbf {r}}_1^2\,{\ddot{\mathbf {r}}_1}^2-\left( \dot{\mathbf {r}}_1\cdot \ddot{\mathbf {r}}_1\right) ^2}}\,. \qquad \dot{~}:=\frac{\mathrm d}{\mathrm d\xi }\,. \end{aligned}$$

In order to check whether a specific matrix $\mathbf {B}$ enables verification of the hypothesized correlation of $U=U_M$ with $\mathbf {r}_1(\xi (\lambda ))=\mathbf{const}.$, it must be shown that this matrix allows for

5 $$\begin{aligned} (\dot{\mathbf {r}}_1)_0=\mathbf {0},\qquad (\ddot{\mathbf {r}}_1)_0=\mathbf {0},\qquad \ldots \,, \end{aligned}$$

where the subscript 0 indicates the onset of loading, *i. e.* $\lambda =0$. Since the eigenvectors represent a complete basis, $\dot{\mathbf {r}}_1$ can be expressed in terms of $\mathbf {r}_j$, with $j=2,3,\ldots ,N$:

6 $$\begin{aligned} \dot{\mathbf {r}}_1=\sum _{j=2}^{N}\,c_{1j}\,\mathbf {r}_j\,, \end{aligned}$$

where

7 $$\begin{aligned} c_{1j}=-\frac{\mathbf {r}_j\cdot \dot{\mathbf {K}}_T\cdot \mathbf {r}_1}{(\chi _j-\chi _1)\,\mathbf {r}_j\cdot \mathbf {B}\cdot \mathbf {r}_j}\,. \end{aligned}$$

Appendix 1 contains the derivation of (7). It would be unfeasible to use (6) for the numerical computation of $\dot{\mathbf {r}}_1$. Instead of doing this, $\dot{\mathbf {r}}_1$ is approximated by a central finite-difference expression. In the following, it will be shown that setting $\mathbf {B}$ as

8 $$\begin{aligned} \mathbf {B}=({\mathbf {K}}_T)_0\,, \end{aligned}$$

where $({\mathbf {K}}_T)_0\equiv {\mathbf {K}}_T$($\xi$($\lambda =0$)) enables $(\dot{\mathbf {r}}_1)_0=\mathbf {0}$. Substitution of (8) into (1) gives

9 $$\begin{aligned} \left[ {\mathbf {K}}_T-\chi _i \,({\mathbf {K}}_T)_0\right] \cdot \mathbf {r}_i=\mathbf {0}\,. \end{aligned}$$

Specialization of (9) for $\lambda =0$ yields

10 $$\begin{aligned} \left[ ({\mathbf {K}}_T)_0-\left( \chi _{i}\right) _0\,({\mathbf {K}}_T)_0\right] \cdot \left( \mathbf {r}_{i}\right) _0=\left( 1-\left( \chi _{i}\right) _0\right) ({\mathbf {K}}_T)_0\cdot \left( \mathbf {r}_{i}\right) _0=\mathbf {0}\qquad \forall i\in \{1,2,\ldots ,N\}\,. \end{aligned}$$

Since $({\mathbf {K}}_T)_0$ is a positive definite matrix,

11 $$\begin{aligned} ({\mathbf {K}}_T)_0\cdot \left( \mathbf {r}_{i}\right) _0\ne \mathbf {0}\,. \end{aligned}$$

Consequently,

12 $$\begin{aligned} \left( \chi _{i}\right) _0=1\,. \end{aligned}$$

Thus, the initial eigenvalues are an *N*-fold eigenvalue, equal to 1. Specialization of (7) for $\lambda =0$ gives

13 $$\begin{aligned} \left( c_{1j}\right) _0=-\frac{\left( \mathbf {r}_j\right) _0\cdot \left( \dot{\mathbf {K}}_T\right) _0\cdot \left( \mathbf {r}_1\right) _0}{\left( \chi _j-\chi _1\right) _0\,\left( \mathbf {r}_j\right) _0\cdot ({\mathbf {K}}_T)_0\cdot \left( \mathbf {r}_j\right) _0}\,. \end{aligned}$$

Differentiation of

14 $$\begin{aligned} \left[ {\mathbf {K}}_T-{\chi _1}\,({\mathbf {K}}_T)_0\right] \cdot \mathbf {r}_1=\mathbf {0}\, \end{aligned}$$

with respect to the chosen parameter yields

15 $$\begin{aligned} \left[ \dot{\mathbf {K}}_T-{{\dot{\chi }}_1}\,({\mathbf {K}}_T)_0\right] \cdot \mathbf {r}_1 +\left[ {{\mathbf {K}}_T}-{{\chi }_1}\,({\mathbf {K}}_T)_0\right] \cdot \dot{\mathbf {r}}_1 =\mathbf {0}\,. \end{aligned}$$

Specialization of (15) for $\lambda =0$ and consideration of

16 $$\begin{aligned} \left[ \left( {{\mathbf {K}}_T}\right) _0-\left( {{\chi _1}}\right) _0\,({\mathbf {K}}_T)_0\right] =\left( 1-\left( \chi _1\right) _0\right) \,\left( {{\mathbf {K}}_T}\right) _0=\mathbf {0} \end{aligned}$$

results in

17 $$\begin{aligned} \left[ \left( \dot{\mathbf {K}}_T\right) _0-\left( {\dot{\chi }}_1\right) _0\,({\mathbf {K}}_T)_0\right] \,\left( {\mathbf {r}}_1\right) _0=\mathbf {0}\,. \end{aligned}$$

Substitution of the orthogonality relations

18 $$\begin{aligned} \left( {\mathbf {r}}_j\right) _0\cdot \left( \dot{\mathbf {K}}_T\right) _0\cdot \left( {\mathbf {r}}_1\right) _0=0\,, \end{aligned}$$

which follow from (17), into (13), and consideration of

19 $$\begin{aligned} \left( \chi _j-\chi _1\right) _0=0\, \end{aligned}$$

give

20 $$\begin{aligned} \left( c_{1j}\right) _0&=-\frac{\left( \mathbf {r}_j\right) _0\cdot \left( \dot{\mathbf {K}}_T\right) _0\cdot \left( \mathbf {r}_1\right) _0}{\left( \chi _j-\chi _1\right) _0\, \left( \mathbf {r}_j\right) _0\cdot ({\mathbf {K}}_T)_0\cdot \left( \mathbf {r}_j\right) _0} ={\frac{``0"}{``0"}} \nonumber \\&= -\frac{ \left( \dot{\mathbf {r}}_j\right) _0\cdot \left( \dot{\mathbf {K}}_T\right) _0\cdot \left( \mathbf {r}_1\right) _0 +\left( \mathbf {r}_j\right) _0\cdot \left( \ddot{\mathbf {K}}_T\right) _0\cdot \left( \mathbf {r}_1\right) _0 +\left( \mathbf {r}_j\right) _0\cdot \left( \dot{\mathbf {K}}_T\right) _0 \cdot \left( \dot{\mathbf {r}}_1\right) _0 }{\left( \dot{\chi _j}-\dot{\chi _1}\right) _0\,\left( \mathbf {r}_j\right) _0\cdot ({\mathbf {K}}_T)_0\cdot \left( \mathbf {r}_j\right) _0}\,, \end{aligned}$$

where L’Hôpital’s rule has been used. In general, $\left( c_{1j}\right) _0\ne 0 \Leftrightarrow \left( \dot{\mathbf {r}}_1\right) _0\ne \mathbf{0 }$. As follows from (20), for the special case of $\left( c_{1j}\right) _0=0 \Leftrightarrow \left( \dot{\mathbf {r}}_1\right) _0=\mathbf{0 }$, the following relation is fulfilled:

21 $$\begin{aligned} \left( \dot{\mathbf {r}}_j\right) _0\cdot \left( \dot{\mathbf {K}}_T\right) _0\cdot \left( \mathbf {r}_1\right) _0 +\left( \mathbf {r}_j\right) _0\cdot \left( \ddot{\mathbf {K}}_T\right) _0\cdot \left( \mathbf {r}_1\right) _0=0\,. \end{aligned}$$

Differentiation of (15) with respect to the chosen parameter and specialization of the obtained equation for $\lambda =0$ and $(\dot{\mathbf {r}}_1)_0=\mathbf {0}$, gives

22 $$\begin{aligned} \left[ (\ddot{\mathbf {K}}_T)_0-(\ddot{\chi }_1)_0\,(\mathbf {K}_T)_0\right] \cdot (\mathbf {r}_1)_0=\mathbf {0}\,, \end{aligned}$$

resulting in the orthogonality relations

23 $$\begin{aligned} (\mathbf {r}_j)_0\cdot (\ddot{\mathbf {K}}_T)_0\cdot (\mathbf {r}_1)_0=0 \end{aligned}$$

in addition to the orthogonality conditions

24 $$\begin{aligned} (\mathbf {r}_j)_0\cdot (\mathbf {K}_T)_0\cdot (\mathbf {r}_1)_0=0\,, \end{aligned}$$

following from (1), and to the orthogonality conditions (18).

Computation of the second derivative of (15) with respect to the chosen parameter and specialization of the result for $\lambda =0$, $(\dot{\mathbf {r}}_1)_0=\mathbf {0}$ and $(\ddot{\mathbf {r}}_1)_0=\mathbf {0}$ yields

25 $$\begin{aligned} \left[ (\dddot{\mathbf {K}}_T)_0-(\dddot{\chi }_1)_0\,(\mathbf {K})_0\right] \cdot (\mathbf {r}_1)_0=\mathbf {0}\,, \end{aligned}$$

resulting in the orthogonality relations

26 $$\begin{aligned} (\mathbf {r}_j)_0\cdot (\dddot{\mathbf {K}}_T)_0\cdot (\mathbf {r}_1)_0=0 \end{aligned}$$

in addition to the three aforementioned orthogonality relations. Hence, $(\dot{\mathbf {r}}_1)_0=\mathbf {0}$, $(\ddot{\mathbf {r}}_1)_0=\mathbf {0}$, ...entails

27 $$\begin{aligned} (\mathbf {r}_j)_0\cdot (\dot{\mathbf {K}}_T)_0\cdot (\mathbf {r}_1)_0=0\,,\quad (\mathbf {r}_j)_0\cdot (\ddot{\mathbf {K}}_T)_0\cdot (\mathbf {r}_1)_0=0\,,\quad (\mathbf {r}_j)_0\cdot (\dddot{\mathbf {K}}_T)_0\cdot (\mathbf {r}_1)_0=0\,,\quad \ldots \ . \end{aligned}$$

If $\mathbf {B}$ had alternatively been chosen *e. g.* as the unit matrix $\mathbf {I}$, (17) would have had to be replaced by

28 $$\begin{aligned} \left[ (\dot{\mathbf {K}}_T)_0-({\dot{\chi }}^{*}_1)_0\,\mathbf {I}\right] \cdot (\mathbf {r}^{*})_0+\left[ ({\mathbf {K}}_T)_0-({\chi }^{*}_1)\,\mathbf {I}\right] \cdot (\dot{\mathbf {r}}^{*})_0=\mathbf {0}\,, \end{aligned}$$

where the symbols marked with an asterisk have replaced the corresponding symbols without an asterisk, reserved for $\mathbf {B}=(\mathbf {K}_T)_0$. In this case, $(\dot{\mathbf {r}}_1^*)_0=\mathbf {0}$, $(\ddot{\mathbf {r}}_1^*)_0=\mathbf {0}$, ... just entails

29 $$\begin{aligned} (\mathbf {r}_j^*)_0\cdot (\dot{\mathbf {K}}_T)_0\cdot (\mathbf {r}_1^*)_0=0\,,\quad {(\mathbf {r}_j^*)_0\cdot (\ddot{\mathbf {K}}_T)_0\cdot (\mathbf {r}_1^*)_0=0\,,\quad \ldots }\ . \end{aligned}$$

In Section 4, it will be shown numerically that $\rho _1=0$ correlates with pure stretching and that $\rho _1=1$ correlates with pure bending.

## Convergence studies concerning spatial and ”temporal” discretizations

The linear eigenvalue problem

30 $$\begin{aligned} \left[ {\mathbf {K}}_T-\chi _i \,\mathbf {B}\right] \cdot \mathbf {r}_i=\mathbf {0}\,, \end{aligned}$$

with

31 $$\begin{aligned} \mathbf {B}=({\mathbf {K}}_T)_0\,, \end{aligned}$$

is solved with the help of six different finite beam elements. Information about these elements is given in Appendix 2. To underline the significance of the coefficient matrix $({\mathbf {K}}_T)_0$ in (30), the analysis results are compared with the ones obtained with

32 $$\begin{aligned} \mathbf {B}=\mathbf {I}\,. \end{aligned}$$

The numerical analysis involves spatial discretizations in the framework of the FEM and ”temporal” discretizations resulting from finite-difference approximations of $\dot{\mathbf {r}}_1$ and $\ddot{\mathbf {r}}_1$ appearing in the expression for $\rho _1$, see (5). In order to separate convergence studies concerning these discretizations from the numerical verification of the asserted geometric mechanical synergism, they have been moved forward to this Section. Table 1 refers to a convergence study concerning the dependence of $\rho _1$ on the number of finite elements used for numerical analysis of a thrust-line arch and a beam subjected to pure bending, treated in Section 4.

**Table 1:** Median value of $\rho _1$ for different numbers of finite elements, for $\mathbf {B}=(\mathbf {K}_T)_0$

Number of	$\rho _1$		
Finite elements	Thrust-line arch, B32	Pure bending, B32OS	Pure bending, B32OSH
2	0.04864	0.706	0.9993
5	0.01059	0.629	0.9992
10	0.00576	0.624	0.9991
20	0.00548	0.622	0.9988
50	0.00544	0.622	0.9967
100	0.00543	0.622	0.9896
200	0.00543	0.622	0.9608
500	0.00543	0.622	0.8133
1000	0.00544	0.622	0.5732

The results of the thrust-line arch were obtained by the Abaqus finite element B32. The deviation of $\rho _1$ from the hypothesized value 0 is less than 0.005709 in analyses with more than 20 elements. The beam subjected to pure bending was analyzed by the Abaqus finite elements B32OS and B32OSH. The results obtained by the former did converge, however, not to the hypothesized value 1. The results obtained by the latter are close to 1 up to 50 elements, with the absolute difference smaller than 0.0034. One reason why fewer degrees of freedom result in a value closer to 1 might be that the projection of a curve onto a unit-sphere in an infinite-dimensional frame onto a finite-dimensional frame leads to an increase of $\rho _1$. In the extreme case of only two degrees of freedom, $\rho _1$ becomes equal to the value of 1.

As follows from (4), computation of $\rho _1$ requires knowledge of $\dot{\mathbf {r}}_1$ and $\ddot{\mathbf {r}}_1$. These vectors are approximated by the following central finite-difference expressions:

33 $$\begin{aligned} \dot{\mathbf {r}}_1(\xi )\approx&\ \frac{\mathbf {r}_1(\xi +\Delta \xi )-\mathbf {r}_1(\xi -\Delta \xi )}{2\,\Delta \xi }\,, \end{aligned}$$

34 $$\begin{aligned} \ddot{\mathbf {r}}_1(\xi )\approx&\ \frac{\mathbf {r}_1(\xi +\Delta \xi )-2\,\mathbf {r}_1(\xi )+\mathbf {r}_1(\xi -\Delta \xi )}{\Delta \xi ^2}\,. \end{aligned}$$

Tables 2 and 3 refer to a convergence study concerning the dependence of the first Frenet-radius $\rho _1$ and the second Frenet-curvature $\kappa _2$, respectively, on the size of the load step $\Delta \lambda$. $\kappa _2$ ($\kappa _2$ is part of the expression for ${\dot{\rho }}_1$, see (39)) is considerably more sensitive to the size of $\Delta \lambda$ than $\rho _1$ because of depending also on the third derivative of $\mathbf {r}_1$, see (41). For the thrust-line arch, analyzed with the Abaqus element B32, the median value of $\kappa _2$ is numerically stable for $\Delta \lambda \ge 0.005$. For pure bending, the median value of $\kappa _2$ for the Abaqus element B32OS is numerically stable for $\Delta \lambda \ge 0.002$.

**Table 2:** Median value of $\rho _1$ for different ”temporal” discretizations, for $\mathbf {B}=(\mathbf {K}_T)_0$

Size of	$\rho _1$	
Load step	Thrust-line arch, B32	Pure bending, B32OS
0.1	0.00521	0.6249
0.05	0.00553	0.6234
0.02	0.00548	0.6225
0.01	0.00546	0.6226
0.005	0.00558	0.6222
0.002	0.00561	0.6220
0.001	0.00561	0.6219

**Table 3:** Median value of $\kappa _2$ for different ”temporal” discretizations, for $\mathbf {B}=(\mathbf {K}_T)_0$

Size of	$\kappa _2$	
Load step	Thrust-line arch, B32	Pure bending, B32OS
0.1	2518	0.934
0.05	795	0.918
0.02	425	0.909
0.01	376	0.914
0.005	393	0.912
0.002	703	0.944
0.001	1513	1.753

## Numerical verification of the asserted geometric-mechanical synergism

### Pure stretching of a two-hinged parabolic arch subjected to a uniformly distributed vertical line load

**Fig. 1:**
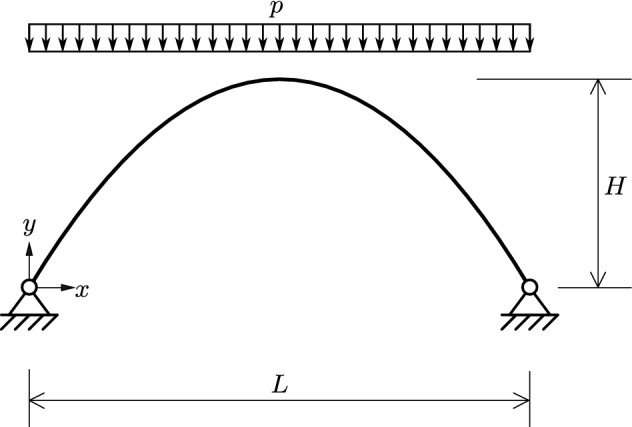
Two-hinged parabolic arch subjected to a uniformly distributed vertical line load

Figure 1 shows a two-hinged parabolic arch of span *L* and height *H*, subjected to a uniformly distributed vertical line load $\lambda \,{\bar{p}}$, where ${\bar{p}}$ denotes the reference load. The values of *L*, *H*, and ${\bar{p}}$ are given as $6\,\text {m}$, $2.4\,\text {m}$, and $8.33\cdot 10^{6}\,\text {N}/\text {m}$, respectively. The initially parabolic arch has a rectangular cross-section. Its height, *h*, is $0.2\,\text {m}$, and its width, *b*, is $0.1\,\text {m}$. The modulus of elasticity, *E*, and Poisson’s ratio, $\nu$, are given as $200\cdot 10^{9}\,\text {Pa}$ and 0.3, respectively. Although the structure is not a three-hinged arch, the bending moments produced by the given loading are small. Hence, the structure may approximately be considered as a thrust-line arch.

The numerical investigation of the arch was performed with six different finite beam elements. Details of these elements are given in Appendix 2. The convergence study, documented in Table 1, has shown that the discretization with 20 finite elements provides sufficiently accurate results. Therefore, further analyses were performed with 20 finite elements. Preliminary analyses, see Table 1, have shown that this discretization provides sufficiently accurate results. Loss of stability of the arch by flexural bifurcation buckling, characterized by $\det \left( {\mathbf {K}}_T\right) =0$ and ${\dot{\lambda }}>0$, occurred at ${p=p_S\approx 2.77\cdot 10^{6}\,\text {N}/\text {m}}$. The existence of a stability limit is relevant to this work only insofar as the choice of the coefficient matrix $\mathbf {B}$ should not have a significantly larger influence on the solution for the radius of the first Frenet-curvature at, and in the vicinity of, the point on the surface curve on the *N*-dimensional unit hypersphere that corresponds to the stability limit than on the remaining part of this curve.

Following the formulation given in Section 2, the eigenvalue problem (1) was solved for $\mathbf {B}=({\mathbf {K}}_T)_0$ as well as for $\mathbf {B}=\mathbf {I}$. According to the hypothetically asserted geometric-mechanical synergism, pure stretching should correlate with $\rho _1=0$.

**Table 4:** Verification (**in green and bold-face**) and falsification (*in red and italics*), respectively, of the hypothetically asserted geometric-mechanical synergism for $\mathbf {B}=({\mathbf {K}}_T)_0$ and $\mathbf {B}=\mathbf {I}$

$\rho _1$	Pi&Bradford	Pi&Bradford	Abaqus	Abaqus	Abaqus	Abaqus
	approximate	accurate	B32	B32H	B31H	B33H
$({\mathbf {K}}_T)_0$	**0.020**$^{\mathrm{a}}$	**0.035**$^{\mathrm{a}}$	**0.024**$^{\mathrm{a}}$	**0.02**$^{\mathrm{a}}$	*0.96*$^{\mathrm{a}}$	*0.08*$^{\mathrm{a}}$
$$\mathbf {I}$$	*0.6*$$^{\mathrm{a}}$$	*0.6*$$^{\mathrm{a}}$$	*0.792*$$^{\mathrm{a}}$$	Undefined$$^{\mathrm{b}}$$	$$\mathbf {10^{-10}}$$$$^{\mathrm{a}}$$	**0.017**$$^{\mathrm{a}}$$

$^{\mathrm{a}}$Maximum value of $\rho _1$

$^{\mathrm{b}}$The first three eigenvalues are constant 3-fold-eigenvalues leading to an indeterminate expression for $\rho _1$

It is seen that for $\mathbf {B}=(\mathbf {K}_T)_0$ the hypothesis is verified for the first four out of the six finite elements considered. Conversely, for $\mathbf {B}=\mathbf {I}$, the hypothesis is verified just for the last two of these elements. The conclusion from the given numerical results that $\mathbf {B}=(\mathbf {K}_T)_0$ is superior to $\mathbf {B}=\mathbf {I}$ would be premature. Nevertheless, the orthogonality of the eigenvectors with respect to $\mathbf {B}=(\mathbf {K}_T)_0$ in addition to the one with respect to $\mathbf {K}_T$ is viewed as an advantage because of leading to an *a priori* known *N*-fold initial eigenvalue $(\chi _i)_0=1$, representing a constraint on $\chi _1(\xi (\lambda ))$, which is the basis for computation of $\rho _1(\xi (\lambda ))$.

**Fig. 2:**
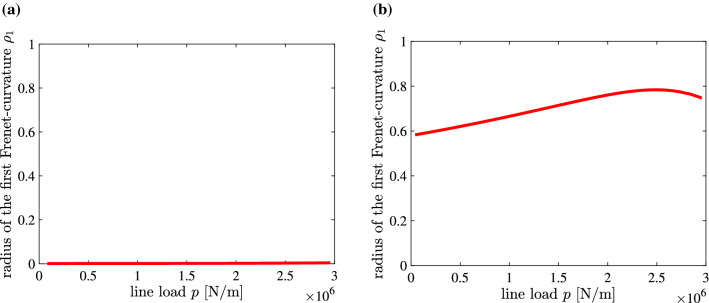
$\rho _1-p$ diagrams obtained with (**a**) $\mathbf {B}=({\mathbf {K}}_T)_0$ and (**b**) $\mathbf {B}=\mathbf {I}$, and with 20 Abaqus-elements B32

Figure 2 shows $\rho _1-p$ diagrams, obtained with $\mathbf {B}=({\mathbf {K}}_T)_0$ and $\mathbf {B}=\mathbf {I}$ and with the Abaqus-element B32. Apart from small deviations of $\rho _1$ from 0, ranging from 0.0011 to 0.17, $\mathbf {B}=({\mathbf {K}}_T)_0$ provides the correct result. The upper bound, $\rho _1=0.17$, refers to a load level well above the stability limit, and out of the region of *p* in Fig. 2a. This confirms the main theoretical finding, reported in Section 2, that $\mathbf {B}=({\mathbf {K}}_T)_0$ enables $\mathbf {r}_1=\mathbf{const}.$, which is the basis for the hypothesized geometric-mechanical synergism of $\rho _1=0$ and $U=U_M$.

### Pure bending of a simply supported beam subjected to equal bending moments at both ends

**Fig. 3:**
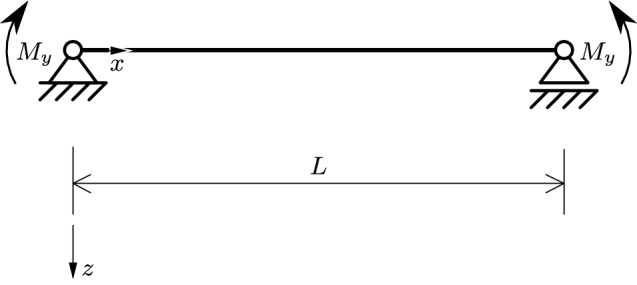
Simply supported beam subjected to equal bending moments at both ends

Figure 3 shows a simply supported beam of length $L=5\,\text {m}$. The IPE 400 beam is subjected to bending moments $\lambda \,{\bar{M}}_y$, with ${\bar{M}}_y=500\,\text {kNm}$, at both ends. The values of *E*, $\nu$, and of the second moment of area, $I_y$, are $210\cdot 10^{9}\,\text {Pa}$, 0.3, and $220\cdot 10^{-6}\,\text {m}^4$, respectively.

The numerical investigation of the beam was performed with the same finite elements that were used for the analysis of the thrust-line arch. The convergence study, documented in Table 1, has shown that the discretization with 20 finite elements provides sufficiently accurate results. Loss of stability of the beam by flexural-torsional buckling, characterized by $\det \left( {\mathbf {K}}_T\right) =0$, occurred at $M_y=M_{y,S}\approx 286\cdot 10^{3}\,\text {N}\,\text {m}$.

The linear eigenvalue problem (1) was solved with $\mathbf {B}=({\mathbf {K}}_T)_0$ as well as with $\mathbf {B}=\mathbf {I}$. According to the hypothetically asserted geometric-mechanical synergism, $U_M=0$ should correlate with $\rho _1=1$. Table 5 refers to verification and falsification, respectively, of this assertion for the two coefficient matrices $\mathbf {B}$ and the six finite beam elements described in Appendix 2.

**Table 5:** Verification (**in green and bold-face**) and falsification (*in red and italics*), respectively, of the hypothetically asserted geometric-mechanical synergism for $\mathbf {B}={({\mathbf {K}}_T)_0}$ and $\mathbf {B}={\mathbf {I}}$

$\rho _1$	Pi&Bradford	Pi&Bradford	Abaqus	Abaqus	Abaqus	Abaqus
	approximate	accurate	B32OS	B32OSH	B31OSH	B33H
$({\mathbf {K}}_T)_0$	*0.19*$^{\mathrm{c}}$	**0.9996**	*0.6041*$^{\mathrm{a}}$	**0.9988**$^{\mathrm{a}}$	**0.9991**$^{\mathrm{a}}$	*0.581*$^{\mathrm{a}}$
$\mathbf {I}$	**0.983**$^{\mathrm{b}}$	**0.980**$^{\mathrm{b}}$	**0.9954**$^{1}$	*0.01*$^{\mathrm{a, d}}$	*0.440*$^{1}$	*0.406*$^{\mathrm{a}}$

$^{\mathrm{a}}$Median value; $^{\mathrm{b}}$Minimum value; $^{\mathrm{c}}$Maximum value; $^{\mathrm{d}}$Numerical issues

**Fig. 4:**
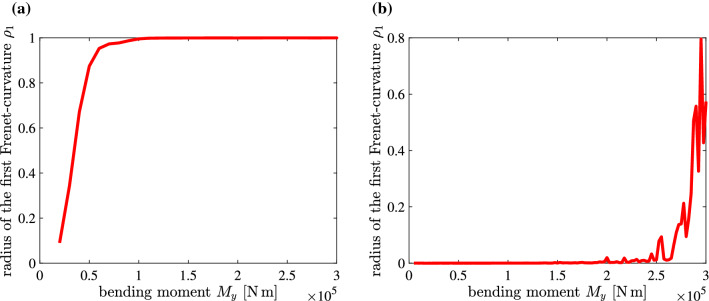
$\rho _1-M_y$ diagrams obtained with (**a**) $\mathbf {B}=({\mathbf {K}}_T)_0$ and (**b**) $\mathbf {B}=\mathbf {I}$, and with the Abaqus element B32OSH

It is seen that for $\mathbf {B}=({\mathbf {K}}_T)_0$ the hypothesis is only verified for the accurate Pi&Bradford element and the Abaqus elements B32OSH and B31OSH. Since the approximate Pi&Bradford element and the Abaqus elements B32OS and B33H provided the correct results for the displacements of the beam and the von Mises stress, membrane locking was ruled out as a possible reason for the falsification of the hypothesis with these three elements. The fact that the Abaqus element B32OSH is an expansion of the Abaqus element B32OS, characterized by additional degrees of freedom to avoid volumetric locking for materials with values of Poisson’s ratio larger than 0.4999999 [1, p. 357], see Appendix 2, might be the reason for its success in the given case, although the value of Poisson’s ratio was chosen as 0.3. A counterargument to the assumed significance of avoiding volumetric locking is the failure of the hypothesis for the Abaqus element B32OSH in case of $\mathbf {B}=\mathbf {I}$. Interestingly, for that case, both Pi&Bradford elements and the Abaqus element B32OS provide the correct result. Figure 4 shows $\rho _1-M$ diagrams obtained with $\mathbf {B}=({\mathbf {K}}_T)_0$ and $\mathbf {B}=\mathbf {I}$ and with the Abaqus element B32OSH.

**Fig. 5:**
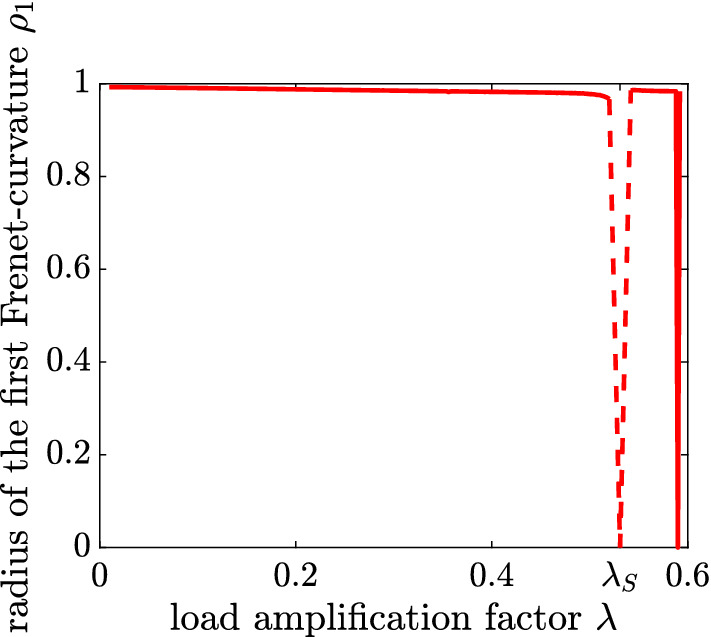
$\rho _1-\lambda$ diagram obtained with the CLE [8], taken from [8]; (the dashed line connects the last computed value with the incorrect theoretical value $\rho _1(\lambda _S)=0$)

In order to demonstrate that the existence of a stability limit may affect the solution for $\rho _1$ in case of an inappropriate choice of the coefficient matrix $\mathbf {B}$, the $\rho _1-\lambda$ diagram obtained with the CLE, *i. e.* with the variable, symmetric, indefinite matrix $\mathbf {B}=-{\dot{{\mathbf {K}}}}_T$, is shown in Fig. 5.

The reason why the hypothesis fails at the stability limit is shown hereafter. Analogous to the derivation of (7), see Appendix 1, the coefficient $c_{1j}$ of

35 $$\begin{aligned} \dot{\mathbf {r}}_1=\sum _{j=2}^N\,c_{1j}\,\mathbf {r}_j\, \end{aligned}$$

is obtained as [8]

36 $$\begin{aligned} c_{1j}={\chi _1}\,\frac{\mathbf {r}_j\,\ddot{{\mathbf {K}}}_T\,\mathbf {r}_1}{(\chi _1-\chi _j)\,\mathbf {r}_j\,{\dot{{\mathbf {K}}}}_T\,\mathbf {r}_j}\,. \end{aligned}$$

Because of $\chi _1=0$ at $\lambda =\lambda _S$,

37 $$\begin{aligned} \dot{\mathbf {r}}_1(\lambda =\lambda _S)=\mathbf {0}\,, \end{aligned}$$

resulting in

38 $$\begin{aligned} \rho _1(\lambda =\lambda _S)=0 \end{aligned}$$

instead of 1.

## Numerical falsification of the hypothetically extended range of validity of the geometric-mechanical synergism

The range of the geometric-mechanical synergism was hypothetically extended to a variable ratio $({U-U_M})/U$. It has been hypothesized that the ratio $({U-U_M})/U$, depending on the load level, is equal to a variable radius of the first Frenet-curvature, $\rho _1$. This calls for a mathematical expression for the rate of change of $\rho _1$, which is given as

39 $$\begin{aligned} {\dot{\rho }}_1=-\dot{s}_1\,\kappa _{2}\,(\mathbf {r}_1\!\cdot \!\mathbf {e}_{3})\,. \end{aligned}$$

Appendix 3 contains the derivation of (39). In this relation,

40 $$\begin{aligned} \dot{s}_1=\left\| \dot{\mathbf {r}}_1\right\| \end{aligned}$$

denotes the speed of a fictitious particle, moving along the curve on the surface of the *N*-dimensional unit hypersphere, described by the vector $\mathbf {r}_1(\xi (\lambda ))$, $\kappa _{2}\,\mathbf {e}_{3}$ is a vector of length $\kappa _{2}$ in the direction of the binormal vector $\mathbf {e}_{3}$, which is a unit vector; $\kappa _{2}$ stands for the second Frenet-curvature [2, 5] of the surface curve; $\kappa _{2}\,\mathbf {e}_{3}$ is given as [2, 5, 14]

41 $$\begin{aligned} \kappa _{2}\,\mathbf {e}_{3}=\frac{\kappa _{1}\,\mathbf {r}_1'''-\kappa _{1}'\,\mathbf {r}_1''}{\kappa ^2_{1}}+\mathbf {r}_1',\qquad ':=\frac{\mathrm d}{{\mathrm d}{{{s_1}}}}\,. \end{aligned}$$

In (41), $\kappa _{1}=1/{\rho _1}$, with $\kappa _{1}$ denoting the first Frenet-curvature of the surface curve, and

42 $$\begin{aligned} \mathbf {r}_1'=\frac{\dot{\mathbf {r}}_1}{\dot{s}_1},\qquad \mathbf {r}_1''=\frac{1}{\dot{s}^2_1}\left( \ddot{\mathbf {r}}_1-\frac{\ddot{s}_1}{\dot{s}_1}\,\dot{\mathbf {r}}_1\right) ,\qquad \mathbf {r}_1'''=\frac{1}{\dot{s}^3_1}\left[ \dddot{\mathbf {r}}_1-3\,\frac{\ddot{s}_1}{\dot{s}_1}\,\ddot{\mathbf {r}}_1+\left( 3\,\frac{\ddot{s}^2_1}{\dot{s}^2_1}-\frac{\dddot{s}_1}{\dot{s}_1}\right) \,\dot{\mathbf {r}}_1\right] \,, \end{aligned}$$

with $\dot{s}_1$ according to (40) and

43 $$\begin{aligned} \ddot{s}_1=\frac{\dot{\mathbf {r}}_1\cdot \ddot{\mathbf {r}}_1}{\dot{s}_1}, \qquad \dddot{s}_1=\frac{\left( \ddot{\mathbf {r}}_1\cdot \ddot{\mathbf {r}}_1+\dot{\mathbf {r}}_1\cdot \dddot{\mathbf {r}}_1\right) \,\dot{s}_1-\left( \dot{\mathbf {r}}_1\cdot \ddot{\mathbf {r}}_1\right) \,\ddot{s}_1}{\dot{s}^2_1}\,. \end{aligned}$$

In the subsequent numerical investigation, $\dot{\mathbf {r}}_1$ and $\ddot{\mathbf {r}}_1$ are approximated by the central finite-difference expressions (33) and (34); $\ddot{\mathbf {r}}_1$ is approximated by the central finite-difference expression

44 $$\begin{aligned} \dddot{\mathbf {r}}_1(\xi _1)\approx \frac{\mathbf {r}_1(\xi _1+2\,\Delta \xi _1)-2\,\mathbf {r}_1(\xi _1+\Delta \xi _1)+2\,\mathbf {r}_1(\xi _1-\Delta \xi _1)-\mathbf {r}_1(\xi _1-2\,\Delta \xi _1)}{2\,\Delta \xi _1^3}\,. \end{aligned}$$

The two examples presented in Section 4 are characterized by

45 $$\begin{aligned} \frac{U-U_M}{U}=\text {const.},\qquad \rho _1\approx \text {const.}\,.\quad \Longrightarrow \quad \left| { \dot{s}}\,\kappa _{2}\,\left( \mathbf {r}_1\!\cdot \!\mathbf {e}_{3}\right) \right| \approx 0\,. \end{aligned}$$

The condition for an extreme value of $\rho _1$, for which $\kappa _2\ne 0$, is given as

46 $$\begin{aligned} \mathbf {r}_1\!\cdot \!\mathbf {e}_{3}=0\,. \end{aligned}$$

**Fig. 6:**
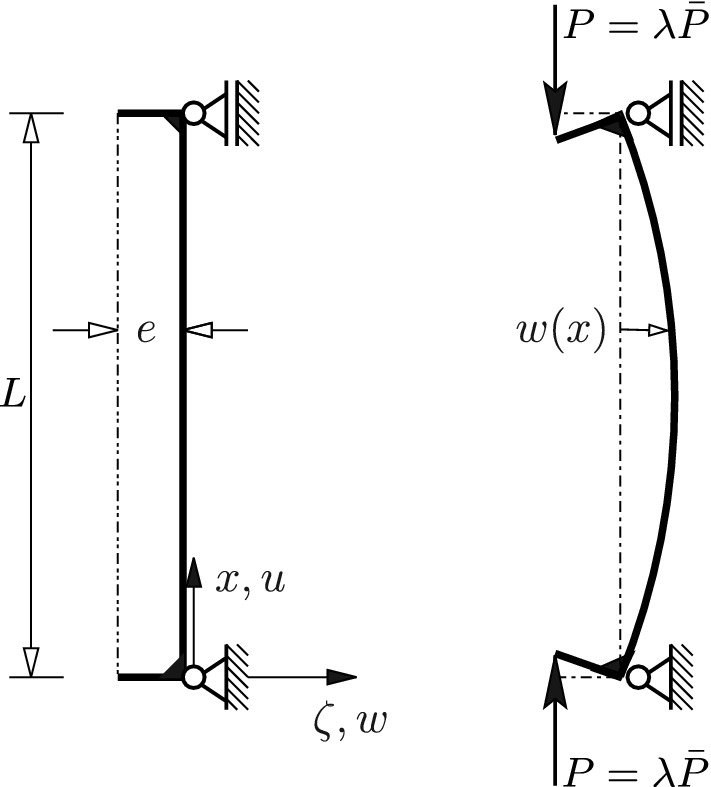
Bar subjected to eccentric compression

Figure 6 shows a bar of length $5\,\text {m}$. The IPE 400 bar is subjected to an eccentric force $\lambda \,{\bar{P}}$, with ${{\bar{P}}=1\,\text {kN}}$ as the reference compressive force. The eccentricity *e*, *E*, and $\nu$ were chosen as ${40.447\cdot 10^{-3}\,\text {m}}$, ${210\cdot 10^{9}\,\text {Pa}}$ and 0.3, respectively.

With the exception of $({U-U_M})/U$ at $\lambda =0$, for which $2^{nd}$-order theory provides the correct result, the given problem must be solved by a full nonlinear analysis, occasionally called $3^{rd}$-order theory [7], for which an analytic solution does not exist. The initial value of $({U-U_M})/U$ is obtained by specializing the general solution for this ratio by means of $2^{nd}$-order theory for $\lambda =0$. This yields

47 $$\begin{aligned} \frac{U_B}{U_M}=\frac{\frac{(N_x\,e)^2}{EI_\zeta }}{\frac{N_x^2}{EA}}=\frac{e^2\,A}{I_\zeta }\quad \Leftrightarrow \quad \frac{U-U_M}{U}=\frac{e^2\,A}{e^2\,A+I_\zeta }\,, \qquad e=\frac{M_\zeta }{N_x}\,, \end{aligned}$$

where *A*, given as $8.0678\cdot 10^{-3}\,\text {m}^2$, stands for the area of the cross-section, and $I_\zeta$, given as ${13.1985\cdot 10^{-6}}\,\text {m}^4$, denotes the second moment of area with respect to the cross-sectional axis $\zeta$. The value of the eccentricity of the compressive force was chosen such that $({U-U_M})/U$ at $\lambda =0$ is equal to 0.5. This resulted in ${e=4.0447\,\text {cm}}$. Table 6 shows that the initial values of $\rho _1$, obtained with $\mathbf {B}=(\mathbf {K}_T)_0$ and $\mathbf {B}=\mathbf {I}$, with four different finite elements each, do not agree with the initial value of ${(U-U_M)}/U$.

**Table 6:** Initial values of $\rho _1$ obtained with $\mathbf {B}=(\mathbf {K}_T)_0$ and $\mathbf {B}=\mathbf {I}$, with four different finite elements each

$(\rho _1)_0$	Abaqus	Abaqus	Abaqus	Abaqus
	B32OS	B32OSH	B31OSH	B33H
$({\mathbf {K}}_T)_0$	0.12	0.003	0.0007	0.0008
$\mathbf {I}$	0.11	0.001	0.97	undef $^{\mathrm{a}}$

$^{\mathrm{a}}$The first three eigenvalues are 3-fold-eigenvalues, leading to an indeterminate expression for $\rho _1$

**Fig. 7:**
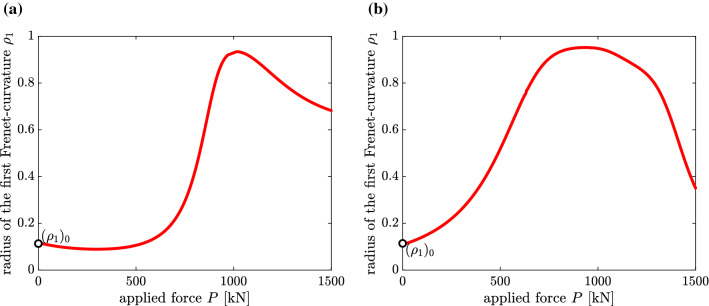
$\rho _1-P$ diagrams obtained with (**a**) $\mathbf {B}=({\mathbf {K}}_T)_0$ and (**b**) $\mathbf {B}=\mathbf {I}$, and the Abaqus element B32OS

Irrespective of the incorrect initial values of $\rho _1$, the course of the functions $\rho _1(P)$, where $P=\lambda \,{\bar{P}}$, will be discussed in the following. Figure 7 shows that $\rho (P)$ is a non-monotonic function. This correlates with the expected non-monotony of the ratio $({U-U_M})/U$, indicating that initially the bending energy is increasing more strongly than the membrane energy. After reaching its maximum value, $\rho (P)$ is decreasing. This correlates with the decrease of $({U-U_M})/U$, indicating that after reaching its maximum value the membrane energy is increasing more strongly than the bending energy. The reason for termination of the $\rho -P$ diagram relatively soon after reaching the maximum value of $\rho$ is the irrelevance of the assumption of a linear elastic material for large deformations. Since the value of $\rho$($P=0$) is significantly smaller than the initial value of $({U-U_M})/U$, *i. e.* 0.5, the hypothetically extended range of validity of the geometric-mechanical synergism could not be verified. This result is corroborated by the supposition that the load level of $\rho _{\text {max}}$ does not agree with the one of $(({U-U_M})/U)_{\text {max}}$.

**Fig. 8:**
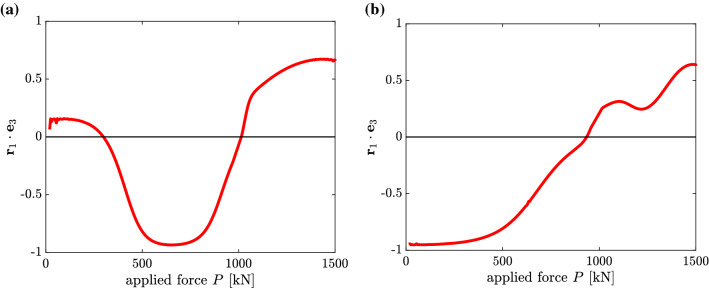
$(\mathbf {r}_1\!\cdot \!\mathbf {e}_3)-P$ diagrams corresponding to the $\rho _1-P$-diagrams shown in Fig. 7

The expression for ${\dot{\rho }}_1$, see (39), refers to a curve on the surface of an *N*-dimensional unit hypersphere. The ($\mathbf {r}_1\!\cdot \!\mathbf {e}_{3}$)$-P$ diagrams shown in Fig. 8 correspond to the $\rho _1-P$ diagrams illustrated in Fig. 7. This proves that (46), *i. e.* $\mathbf {r}_1\!\cdot \!\mathbf {e}_3=0$, is the condition for an extreme value of $\rho _1$.

One attempt to improve the original hypothesis was to replace $\rho _1$ by $\sqrt{1-(\mathbf {r}_1\!\cdot \!\mathbf {e}_3)^2}$. The rationale for this modification was that

48 $$\begin{aligned} \rho _1\le \sqrt{1-(\mathbf {r}_1\!\cdot \!\mathbf {e}_3)^2}\le 1. \end{aligned}$$

Another attempt was to explicitly consider $\kappa _2$ in the hypothesis, albeit without abandoning the restriction of the constancy of the coefficient matrix $\mathbf {B}$ in the linear eigenvalue problem (1). Both attempts were not successful. The second one did not rule out the possibility of values of $\rho _1$ larger than 1, which obviously do not correlate with corresponding values of $({U-U_M})/U$.

## Conclusions

. .

## Appendix 1: Derivation of (7)

Differentiation of

49 $$\begin{aligned} \left[ ({\mathbf {K}}_T)_0-\chi _1\,\mathbf {B}\right] \cdot \mathbf {r}_1=\mathbf {0} \end{aligned}$$

with respect of $\lambda$ gives

50 $$\begin{aligned} \left[ ({\dot{\mathbf {K}}}_T)-{\dot{\chi }}_1\,\mathbf {B}\right] \cdot \mathbf {r}_1+\left[ ({\mathbf {K}}_T)_0-\chi _1\,\mathbf {B}\right] \cdot \dot{\mathbf {r}}_1=\mathbf {0}\,. \end{aligned}$$

Substitution of

51 $$\begin{aligned} \dot{\mathbf {r}}_1=\sum _{k=2}^N c_{1k}\,\mathbf {r}_k \end{aligned}$$

into (50), followed by premultiplication of the resulting relation by $\mathbf {r}_j, j\ne 1$, yields

52 $$\begin{aligned} \mathbf {r}_j\cdot \left[ \dot{\mathbf {K}}_T-{\dot{\chi }}_1\,\mathbf {B}\right] \cdot \mathbf {r}_1+\mathbf {r}_j\cdot \left[ \mathbf {K}_T-\chi _1\,\mathbf {B}\right] \cdot \sum _{k=2}^N c_{1k}\,\mathbf {r}_k={0}. \end{aligned}$$

Making use of the orthogonality relations

53 $$\begin{aligned} \mathbf {r}_j\cdot \mathbf {B}\cdot \mathbf {r}_1=0,\quad j\ne 1 \end{aligned}$$

and

54 $$\begin{aligned} \mathbf {r}_j\cdot \mathbf {K}_T\cdot \mathbf {r}_k=0,\quad \mathbf {r}_j\cdot \mathbf {B}\cdot \mathbf {r}_k=0,\quad k\ne j, \end{aligned}$$

results in

55 $$\begin{aligned} c_{1j}=-\frac{\mathbf {r}_j\cdot \dot{\mathbf {K}}_T\cdot \mathbf {r}_1}{\mathbf {r}_j\cdot \left[ \mathbf {K}_T-\chi _1\mathbf {B}\right] \cdot \mathbf {r}_j}. \end{aligned}$$

Premultiplication of

56 $$\begin{aligned} \left[ \mathbf {K}_T-\chi _j\mathbf {B}\right] \cdot \mathbf {r}_j=\mathbf {0} \end{aligned}$$

by $$\mathbf {r}_j$$ yields

57 $$\begin{aligned} \mathbf {r}_j\cdot \mathbf {K}_T\cdot \mathbf {r}_j=\chi _j\,\mathbf {r}_j\cdot \dot{\mathbf {K}}_T\cdot \mathbf {r}_j\,. \end{aligned}$$

Substitution of (57) into (55) gives

58 $$\begin{aligned} c_{1j}=-\frac{\mathbf {r}_j\cdot \dot{\mathbf {K}}_T\cdot \mathbf {r}_1}{(\chi _j-\chi _1)\,\mathbf {r}_j\cdot \mathbf {B}\cdot \mathbf {r}_j}\,. \end{aligned}$$

## Appendix 2: Details of the finite beam elements used in the numerical investigation

**Table 7:** Finite elements used in the numerical investigation

Name	Formulation	Nodes	Nr. of d.o.f.
Pi&Bradford - approx. [10–13]	Displ., Euler-Bernoulli	2/cubic	14 + 2
Pi&Bradford - accurate [10–13]	Displ., Euler-Bernoulli	2/cubic	14 + 2
Abaqus B32 [1]	Displ., Timoshenko	3/quad.	18
Abaqus B32OS [1]	Displ., Timoshenko, open section	3/quad.	21
Abaqus B32H [1]	Hybrid, Timoshenko	3/quad.	18 + 6
Abaqus B32OSH [1]	Hybrid, Timoshenko, open section	3/quad.	21 + 6
Abaqus B31H [1]	Hybrid, Timoshenko	2/linear	12 + 3
Abaqus B31OSH [1]	Hybrid, Timoshenko, open section	2/linear	14 + 3
Abaqus B33H [1]	Hybrid, Bernoulli	2/cubic	12 + 6 + 6

## Appendix 3: Derivation of (39)

For convenience’s sake and because of the fact that this derivation is not restricted to a particular eigenvector, the subscript “1” will be omitted in the following.

Differentiation of $\mathbf {r}\cdot \mathbf {r}=1$ with respect to the chosen parameter gives

59 $$\begin{aligned} \mathbf {r}\cdot \dot{\mathbf {r}}=0\,. \end{aligned}$$

Substitution of

60 $$\begin{aligned} \dot{\mathbf {r}}=\dot{s}\,\mathbf {e}_1\,, \end{aligned}$$

where $\mathbf {e}_1$ denotes the first vector of the Frenet frame, which is a unit vector (76,77), into (59) yields

61 $$\begin{aligned} \dot{s}\,\left( \mathbf {r}\!\cdot \!\mathbf {e}_1\right) =0 \end{aligned}$$

where

62 $$\begin{aligned} \mathbf {r}\!\cdot \!\mathbf {e}_1=0\,. \end{aligned}$$

Differentiation of (59) and (60) with respect to the chosen parameter results in

63 $$\begin{aligned} \dot{\mathbf {r}}\cdot \dot{\mathbf {r}}+{\mathbf {r}}\cdot \ddot{\mathbf {r}}=0 \end{aligned}$$

and

64 $$\begin{aligned} \ddot{\mathbf {r}}=\ddot{s}\,\mathbf {e}_1+\dot{s}\,\dot{\mathbf {e}}_1\,, \end{aligned}$$

respectively, where, according to (74) and (79),

65 $$\begin{aligned} \dot{\mathbf {e}}_1=\dot{s}\,\frac{\mathbf {e}_2}{\rho }\,, \end{aligned}$$

with $\mathbf {e}_2$ denoting the second vector of the Frenet frame, which is a unit vector (76,77). Substitution of (60) and (64) into (63) and consideration of (65) gives

66 $$\begin{aligned} \dot{s}^2+\mathbf {r}\cdot \left( \ddot{s}\,\mathbf {e}_1+\frac{\dot{s}^2}{\rho }\,\mathbf {e}_2\right) =0\,. \end{aligned}$$

Consideration of (62) results in

67 $$\begin{aligned} \dot{s}^2\left( 1+\frac{1}{\rho }\,\mathbf {r}\cdot \mathbf {e}_2\right) =0 \end{aligned}$$

where

68 $$\begin{aligned} \rho =-\mathbf {r}\!\cdot \!\mathbf {e}_2\,. \end{aligned}$$

Differentiation of (68) with respect to the parameter yields

69 $$\begin{aligned} {\dot{\rho }}=-\left( \mathbf {r}\!\cdot \!\mathbf {e}_2\right) \!\dot{~}=-\left( \dot{\mathbf {r}}\!\cdot \!\mathbf {e}_2+\mathbf {r}\cdot \dot{\mathbf {e}}_2\right) , \end{aligned}$$

where, according to (74),

70 $$\begin{aligned} \dot{\mathbf {e}}_2=\dot{s}\left( -\frac{1}{\rho }\,\mathbf {e}_1+\kappa _2\,\mathbf {e}_{3}\right) \,, \end{aligned}$$

with $\kappa _2$ denoting the second Frenet-curvature, see (78), and $\mathbf {e}_3$ standing for the binormal vector, which is a unit vector (76,77). Substitution of (60) and (70) into (69) gives

71 $$\begin{aligned} {\dot{\rho }}=-\dot{s}\left( \mathbf {e}_1\cdot \mathbf {e}_2+\mathbf {r}\cdot \left( -\frac{1}{\rho }\,\mathbf {e}_1+\kappa _2\,\mathbf {e}_{3}\right) \right) \,. \end{aligned}$$

Consideration of

72 $$\begin{aligned} \mathbf {e}_1\!\cdot \!\mathbf {e}_2=0\,, \end{aligned}$$

following from (75), and of (62) finally yields:

73 $$\begin{aligned} {\dot{\rho }}=-\dot{s}\,\kappa _2\,\left( \mathbf {r}\cdot \mathbf {e}_{3}\right) \,. \end{aligned}$$

## Appendix 4: Frenet formulae in *N* dimensions

The Frenet formulae in *N* dimensions are given as [2, 5, 15]

74 $$\begin{aligned} \begin{pmatrix} \mathbf {e}_1'(s)\\ \mathbf {e}_2'(s)\\ \mathbf {e}_3'(s)\\ \mathbf {e}_4'(s)\\ \vdots \\ \mathbf {e}_{N\text {-}2}'(s) \\ \mathbf {e}_{N\text {-}1}'(s) \\ \mathbf {e}_N'(s) \\ \end{pmatrix} = \Vert \mathbf {r}'(s) \Vert \cdot \begin{bmatrix} 0 &{}\kappa _1(s)&{}0&{}0&{}\cdots &{}0&{}0&{}0\\ -\kappa _1(s)&{}0&{}\kappa _2(s)&{}0&{}\ddots &{}0&{}0&{}0\\ 0&{}-\kappa _2(s)&{}0&{}\kappa _3(s)&{}\ddots &{}0&{}0&{}0\\ 0&{}0&{}-\kappa _3(s)&{}0&{}\ddots &{}0&{}0&{}0\\ \vdots &{}\ddots &{}\ddots &{}\ddots &{}\ddots &{}\ddots &{}\ddots &{}\vdots \\ 0&{}0&{}0&{}0&{}\ddots &{}0&{}\kappa _{N\text {-}2}(s)&{}0 \\ 0&{}0&{}0&{}0&{}\ddots &{}-\kappa _{N\text {-}2}(s)&{}0 &{} \tau (s) \\ 0&{}0&{}0&{}0&{}\cdots &{}0&{} -\tau (s) &{} 0 \\ \end{bmatrix} \cdot \begin{pmatrix} \mathbf {e}_1(s) \\ \mathbf {e}_2(s)\\ \mathbf {e}_3(s)\\ \mathbf {e}_4(s)\\ \vdots \\ \mathbf {e}_{N\text {-}2}(s) \\ \mathbf {e}_{N\text {-}1}(s) \\ \mathbf {e}_N(s) \\ \end{pmatrix}\,,\nonumber \\ \end{aligned}$$

with the unit vectors

75 $$\begin{aligned}&\tilde{\mathbf {e}}_i(s)=\mathbf {r}^{(i)}(s)-\sum _{j=1}^{i-1}\left( \mathbf {r}^{(i)}(s)\!\cdot \!\mathbf {e}_j(s)\right) \,\mathbf {e}_j(s)\qquad \forall i=\{1,...,N\text {-}1\}\,, \end{aligned}$$

76 $$\begin{aligned}&{\mathbf {e}}_i(s)=\frac{\tilde{\mathbf {e}}_i(s)}{\left\| \tilde{\mathbf {e}}_i(s)\right\| }\,, \end{aligned}$$

77 $$\begin{aligned}&\mathbf {e}_N(s)=\mathbf {e}_1(s)\times \mathbf {e}_2(s)\times \dots \times \mathbf {e}_{N\text {-}2}(s)\times \mathbf {e}_{N\text {-}1}(s)\,, \end{aligned}$$

and the generalized curvatures defined as

78 $$\begin{aligned} \kappa _i(s)=\frac{\mathbf {e}'_i(s)\cdot \mathbf {e}_{i+1}(s)}{\Vert \mathbf {r}'(s) \Vert }\,. \end{aligned}$$

All curvatures except the top curvature, *i. e.* the torsion $\tau$, are non-negative reals [2].

The radius of the first curvature, $\rho _1(s)$, is the inverse of the first curvature $\kappa _1(s)$:

79 $$\begin{aligned} \rho _1(s)=\frac{1}{\kappa _1(s)}. \end{aligned}$$

## Appendix 5: List of symbols and abbreviations

**Table 8:** Nomenclature

*a*	$[\text {rad}]$	Acceleration of a fictitious particle moving along a curve on the unit sphere [6]
*b*	$[\text {m}]$	Width of the beam
$c_{1j}$	$[-]$	Components of the objective eigenvector-space of $\dot{\mathbf {r}}_1$, defined in (6)
*e*	$[\text {m}]$	Eccentricity of the normal force
$\mathbf {e}_1$		Tangent vector of the Frenet frame, defined in (64)
$\mathbf {e}_2$		Normal vector of the Frenet frame, defined in (64)
$\mathbf {e}_{3}$		Binormal vector of the Frenet frame, defined in (41)
*h*	$[\text {m}]$	Height of the beam
*k*	$[\text {N}\,\text {m}]$	Proportionality coefficient in the mathematical formulation of a hypothesis for $(U-U_M)/U$ [6]
$\ell$	$[\text {m}]$	Length of a bar
*p*	$[\text {N}/\text {m}]$	Vertical line load of the parabolic two-hinged arch, see Fig. 1
$p_S$	$[\text {N}/\text {m}]$	Vertical line load at the stability limit of the parabolic two-hinged arch
$\mathbf {q}$	$[\text {m}],[\text {rad}]$	Vector of nodal degrees of freedom in the framework of the FEM, introduced in (3)
$\mathbf {r}$	$[\text {m}],[\text {rad}]$	Orthonormal unit-eigenvector of the eigenvalue-problem (1)
*s*	$[-]$	Arc-length of the curve on the surface of the unit sphere, described by the normalized fundamental eigenvector, introduced in (40)
*A*	$[\text {m}^2]$	Area of the cross section of the beam, used in (47)
$\mathbf {B}$	Various	Constant, symmetric, positive definite coefficient matrix, introduced in (1)
*E*	$[\text {N}/\text {m}^2]$	Modulus of elasticity
*I*	$[\text {m}^4]$	Relevant principal second moment of area of the cross-section of a bar, used in (47)
$\mathbf {I}$	$[-]$	Identity matrix, introduced in (32)
${\mathbf {K}}_T$	$[\text {N}/\text {m}], [\text {N}\,\text {m}/\text {rad}]$	Tangent stiffness matrix, defined in (3)
$({\mathbf {K}}_T)_0$	$[\text {N}/\text {m}], [\text {N}\,\text {m}/\text {rad}]$	Tangent stiffness matrix in the unloaded configuration, introduced in (8)
*N*	$[-]$	Number of degrees of freedom in the FEM-simulation, after applying the boundary conditions, with *N*>2
$N_x$	$[\text {N}]$	Normal force, used in (47)
*M*	$[\text {N}\,\text {m}]$	Bending moment applied at both ends, see Fig. 3
$P$	$[\text {N}]$	Force applied at both ends, see Fig. 6
$\mathbf {P}$	$[\text {N}],[\text {N}\,\text {m}]$	Vector of work-equivalent node forces, defined in (3)
$\bar{\mathbf {P}}$	$[\text {N}],[\text {N}\,\text {m}]$	Reference vector of work-equivalent node forces, defined in (3) as $\bar{\mathbf {P}}=\mathbf {P}/\lambda$
*U*	$[\text {N}\,\text {m}]$	Strain energy
$U_B$	$[\text {N}\,\text {m}]$	Bending energy
$U_M$	$[\text {N}\,\text {m}]$	Stretching (membrane) energy
$\kappa _1$	$[-]$	First Frenet-curvature
$\kappa _2$	$[-]$	Second Frenet-curvature
$\lambda$	$[-]$	Amplification factor of the reference load
$\xi$	$[\text {m}]$	Arc length of the FEM-displacements, defined in (1)
$\rho$	$[-]$	Radius of the first Frenet-curvature of $\mathbf {r}$, defined in (1)
$\tau$	$[-]$	Torsion: $\kappa _{N\text {-}1}$, representing the last curvature [2, 5], introduced in (74)
$\chi$	Various	Eigenvalue in (1)
